# Community-based health care for indigenous women in Mexico: a qualitative evaluation

**DOI:** 10.1186/1475-9276-13-2

**Published:** 2014-01-06

**Authors:** Blanca Pelcastre-Villafuerte, Myriam Ruiz, Sergio Meneses, Claudia Amaya, Margarita Márquez, Arianna Taboada, Katherine Careaga

**Affiliations:** 1 Center for Health Systems Research, National Institute of Public Health, Universidad No. 655 Colonia Santa María Ahuacatitlán, Cerrada Los Pinos y Caminera, Cuernavaca, Morelos C.P. 62100, Mexico; 2 School of Medicine, Department of Public Health, Industrial University of Colombia, Cra 27 Calle 9 Ciudad Universitaria, Bucaramanga, Santander PBX 634400, Colombia; 3 Regional Center for Public Health Research, National Institute of Public Health, 19 Poniente Esquina 4ª Norte s/n, Colonia Centro, Tapachula, Chiapas C.P. 30700, Mexico; 4 Center for Research in Nutrition and Health, National Institute of Public Health, Universidad No. 655 Colonia Santa María Ahuacatitlán, Cuernavaca, Morelos C.P. 62100, Mexico; 5, Lote 4 Mza 273 Calle 21 entre 65 y 70 Sur Colonia Ejido, Playa del Carmen, Quintana Roo C.P. 77780, Mexico; 6 Biology Department, Fort Lewis College, 1000 Rim Drive, Durango, CO 81301, USA

**Keywords:** Indigenous women, Health disparities, Cultural competency, Qualitative evaluation

## Abstract

**Introduction:**

Indigenous women in Mexico represent a vulnerable population in which three kinds of discrimination converge (ethnicity, gender and class), having direct repercussions on health status. The discrimination and inequity in health care settings brought this population to the fore as a priority group for institutional action. The objective of this study was to evaluate the processes and performance of the “*Casa de la Mujer Indígena*”, a community based project for culturally and linguistically appropriate service delivery for indigenous women. The evaluation summarizes perspectives from diverse stakeholders involved in the implementation of the model, including users, local authorities, and institutional representatives.

**Methods:**

The study covered five *Casas* implementation sites located in four Mexican states. A qualitative process evaluation focused on systematically analyzing the *Casas* project processes and performance was conducted using archival information and semi-structured interviews. Sixty-two interviews were conducted, and grounded theory approach was applied for data analysis.

**Results:**

Few similarities were observed between the proposed model of service delivery and its implementation in diverse locations, signaling discordant operating processes. Evidence gathered from *Casas* personnel highlighted their ability to detect obstetric emergencies and domestic violence cases, as well as contribute to the empowerment of women in the indigenous communities served by the project. These themes directly translated to increases in the reporting of abuse and referrals for obstetric emergencies.

**Conclusions:**

The model’s cultural and linguistic competency, and contributions to increased referrals for obstetric emergencies and abuse are notable successes. The flexibility and community-based nature of the model has allowed it to be adapted to the particularities of diverse indigenous contexts. Local, culturally appropriate implementation has been facilitated by the fact that the *Casas* have been implemented with local leadership and local women have taken ownership. Users express overall satisfaction with service delivery, while providing constructive feedback for the improvement of existing *Casas*, as well as more cost-effective implementation of the model in new sites. Integration of user’s input obtained from this process evaluation into future planning will undoubtedly increase buy-in. The *Casas* model is pertinent and viable to other contexts where indigenous women experience disparities in care.

## Introduction

Indigenous^a^ populations are among the most marginalized groups in the world. Due to social marginalization, indigenous people are more likely to be poor, have fewer years of formal education, and less access to health services [1,2]. Furthermore, dominant culture’s lack of understanding of indigenous populations surfaces in discriminatory, racist, and exclusionary practices and attitudes [3] The indigenous populations of Mexico – the largest on the American continent^b^[4]–, are not exempt from conditions of poverty and marginalization. 75.6% of the indigenous population lives in locations classified as high or very high marginalization; 22% live in homes with dirt floors; 26% do not have potable water and 45.6% lack indoor plumbing [4]. Being indigenous also corresponds to disparities in national health indicators. Life expectancy at birth of indigenous Mexicans is eight or nine years less than the national average. Likewise, infant mortality rates among the indigenous are twice the national average [5].

Indigenous women constitute a subgroup of the Mexican indigenous population with the highest lag in health status. As three kinds of discrimination converge - ethnic, gender and class – there is a unique matrix of ailments and diseases correlating to this triple subordination [6]. Maternal mortality is a case example of such outcomes, with official data indicating that the major concentration of maternal mortality in Mexico occurs in indigenous and rural areas of the central and southeastern regions of the country [7,8]. Indigenous women’s risk of dying from childbirth is three times higher than for women in the rest of the country [9]. In 2008, the national maternal mortality rate was 53 per 100,000 live births. In the same time period, Guerrero—a state with a significant indigenous population and the lowest social indicators of the country—had a maternal mortality rate of 104 per 100,000 live births [10].

These statistics highlight the problems of inequality, discrimination, and marginalization in which the majority of the Mexican indigenous women live. The aforementioned data on maternal mortality is the result of a particular context where lack of health services, timely care, adequate infrastructure, and economic resources converge with discrimination and cultural gaps between hegemonic Western medicine and indigenous forms of understanding health. The complexity of the situation and the seriousness of resulting health indicators begs for effective public health strategies to address these challenges.

Coverage of public services has been insufficient to properly attend to the special needs of indigenous women. In order to succeed, publically funded women’s health services must take into account geographic, economic and cultural barriers to care, as well as the perceptions on the part of the individual and community about the quality and efficacy of the services [11]. Furthermore, the consideration of indigenous communities’ complex models for health-related decision-making, based on their beliefs about the health-illness/disease/sickness process, impact the ability of non-indigenous institutions and providers to carry out timely, necessary interventions, while ensuring quality and access [12-18]. In Mexico, there has been an historic need to bridge services rooted in Western medical practices with traditional medical knowledge, and in recent years, an increasing recognition of the importance of this unmet need and attempt to address it through the development of intercultural models of public health service delivery.

Among institutional efforts that have been implemented to address the health care needs among indigenous women, the Project *Casa de la Mujer Indígena*, (hereto referred to as *Casas)* stands out. This project brings together the local indigenous community, civil society organizations (NGO’s), and public institutions, in order to create a physical space that serves three primary functions:

1) Provide health education and basic health care delivered by indigenous community health workers or *promotoras*

2) Serve as a link or referral mechanism to mainstream health services, specifically reproductive health care

3) Offer meeting space where *promotoras* and traditional birth attendants (TBAs), medical professionals, as well as non-profit organizations working in health, reproductive rights, and domestic violence issues in indigenous communities, can collaborate on local actions.

The *Casas* project was initiated by the Inter-sectorial Program for Indigenous Women’s Health Care, and overseen by Mexico’s federal Office of Representation for the Development of Indigenous Peoples. This initiative is part of a larger framework of cultural competency being applied in Mexican states to improve health care delivery and patient satisfaction by utilizing local Western health resources and reinforcing traditional medical roles, such as the TBAs. The intent of such a framework is to bridge the cultural distance between institutionalized, hegemonic forms of health care and indigenous forms of understanding and tending to health – especially reproductive health– within each community. The framework is specifically focused on alleviating language differences that impede effective doctor-patient communication about symptoms, pain and expectations, as well as mitigating the distrust of allopathic medicine, particularly in regards to indigenous women and male providers, while ultimately improving the quality of the services offered to indigenous women. The purpose of the study was to conduct an evaluation of processes and performance of the *Casas* from the point of view of different actors involved in their implementation, including users, authorities and representations from other institutions. The driving research question was “How do internal and external stakeholders assess the organizational experience and implementation of the *Casas* model?”

### Study setting

At the time of the study (2008), five *Casas* had been established in four Mexican states: two in Oaxaca (Matías Romero and San Mateo del Mar); one in Chalchihuitán, Chiapas; one in Ometepec, Guerrero and one in Cuetzalan, Puebla [6]. An average of 6 to 8 women work in each *Casa*: a coordinator in charge of administrating resources, forming connections with institutional authorities and organizing the daily activities; advisors, who offer support and orientation concerning the *Casas*; and *promotoras*, TBAs, nurses and attorneys specializing in maternal health care and domestic violence, from the psychological, legal and medical (traditional and Western) standpoints.

The *Casas* are located within traditionally indigenous regions of Mexico, which are characterized by having a high density of indigenous population, being multi-ethnic spaces where various languages are spoken, and experiencing high or very high marginalization, as defined by the national census. Between 36.8% (Oaxaca) to 98.3% (Chiapas) of the indigenous populations in the regions served by the *Casas* experience high or very high marginalization [19]. These are typically rural setting, many of them at least 3 kilometers away from paved roads. This represents a serious barrier in access to supplies, transportation routes, commercialization of local products, emergency health care services, and educational services, all of which hamper the capabilities of institutional programs to operate efficiently [20].

## Methods

### Study design

The study design was a processes evaluation guided by perspectives from diverse stakeholders involved in the implementation of the model such as users, local authorities, and institutional representatives. Two primary techniques were used to collect information and carry out the evaluation: the analysis of official regulatory and program documents and key informant interviews. The study design drew on phenomenology [21] as a theoretical and methodological approach to explore the experience of the participants who carry out diverse functions and duties in the *Casas*, as well as of the indigenous women who have received services. The design was also informed by Patton’s qualitative evaluation approach, which considers informal questions and unexpected consequences within the overall context of the development and implementation of the programs [22]. Additionally, Rossi and colleagues evaluation framework [23] was used to formulate specific questions regarding project operation, structure, organization, processes and results. The multiple theoretical perspectives were purposefully incorporated in the study design in order to evaluate the many care-seeking and health service utilization factors the *Casas* sought to address.

### Sample selection

The criteria for inclusion for the key informants were defined by position and connection to the *Casas,* with four main profiles of interest: (1) coordinators and advisors, (2) operations personnel and collaborating local health care providers (TBAs, promotor as, psychologists, attorneys), (3) women receiving services, and (4) other participants. The latter category included local authorities such as the mayor, those in charge of indigenous affairs, other health care providers, judicial staff, and administrators of public social service agencies. These profiles were selected to gain a close-up understanding of those involved in the *Casas’* core functions.

The participants were recruited through convenience sampling in each of the *Casas*. Prior to recruitment efforts, informational meetings were held with the research team and the coordinators of each facility in order to present and explain the project, facilitate contact with other participants, and to be able to access archival documents that described the creation and operation of the *Casas*. The facility coordinators and research team then developed a mutually agreed-upon schedule for data collection.

### Ethical considerations

The study was approved by the Research and Ethics Commissions of Mexico’s National Institute of Public Health and adheres to the commission’s ethical guidelines for conducting social research with indigenous populations. Each potential participant was informed in detail of the objectives, procedures, risks and benefits of the study and only after assuring her understanding was she invited to participate. Those willing to participate were asked to sign a letter of informed consent, which was in Spanish only. Translators were available to verbally assist in obtaining the informed consent of monolingual indigenous women.

### Data collection

Interview guidelines were designed for each of the four participant types. The semi-structured interview guide employed a narrative approximation technique, which typically began with a recounting of how the *Casas* project had begun and how each interviewee had become involved in the project. Other themes that were explored in the interview were: the mission, functions and structure of the *Casas*, the benefits of the *Casas* for indigenous women, and obstacles to operations and sustainability.

The original *Casas* project proposal and previous evaluation reports were reviewed in detail to understand the basic functions of the *Casas* and design instruments that probed for information not readily available through archival documents. The evaluation process took into consideration the different aspects of *Casas’* daily operations, organization, and administration. Observation guidelines and a questionnaire were designed and applied in all the *Casas* according to the established data collection schedule*.* The field work was conducted between January and June of 2007. Interviews were conducted by members of the research team, all of whom had significant experience in qualitative studies, with the assistance of indigenous translators when the informants were monolingual speakers of an indigenous language. Prior to the field work, the entire team participated in a standardized training in interviewing techniques, with the aim of strengthening the study’s reliability. The translators who supported interviews conducted in an indigenous language also participated voluntarily, and were trained in interview techniques to ensure that the questions asked as well as the information collected were translated accordingly. Furthermore, to protect the confidential nature of the information, all translators were asked to sign a confidentiality agreement.

All interviews were audio-taped, with previous informed consent. The transcript for an interview conducted in an indigenous language was reviewed by the same translator present at the time of the interview. All interviews were transcribed in a format accessible for analysis, which was carried out with the support of the qualitative analysis program Atlas-ti (v.5.2).

The final distribution of the total number of interviews is shown in Table 1.

**Table 1:** Distribution of interviews

**Type of participant**	**Total**
Central coordination	2
General coordinators and advisors	7
Users	14
Local resources and operations personnel	32
Other participants	7
**TOTAL**	62

### Data analysis

Organization of the data followed the steps proposed by grounded theory [24] and incorporated a critical social psychology perspective, which considers discourse as a social practice that constructs social identities [25-27]. The analysis aimed at understanding the participants’ perspectives and highlighting their experiences in relation to the *Casas’* structure, processes, and performance. As such, the categories that guided the interviews permitted the thematic organization of data, using an inductive approach. Each category was examined in relation to 1) primary activities; 2) the main obstacles that the *Casas* face in different contexts; 3) the main benefits for the population and for the health services, as well as 4) the possibility of creating a plan replicable for other indigenous communities. As new concepts and themes emerged, the transcripts were re-examined and the categories refined [28]. The themes that emerged were discussed and reviewed by the research team.

## Results

Guided by the point-of-view of participants and their respective organizations, the *Casas* evaluation captured structural and programmatic elements that result in better access to care for indigenous women. These elements are synthesized below.

### Personnel capacity and accessibility

The staffing of each *Casa* varied widely. It is important to note that although we refer to staff throughout the article, all personnel are volunteers. They receive stipends, but are not salaried. From the perspective of key informants, the women that work in the *Casas* are perceived as active, dedicated, and organized participants in the fight for women’s rights. This perception legitimizes their role in the *Casas*, and they are seen as extremely committed to their work. All the *Casas* have a general coordinator, but none have in-house medical personnel. In Chiapas, San Mateo del Mar and Cuetzalan services are available 24 hours a day, 7 days a week. This is not the case at the other two *Casas* due to lack of personnel, although in emergency situations, staff are called at home and respond at any hour. The TBAs play an important role in the *Casas*. They are natives of their respective regions and speak the corresponding indigenous language. Their availability varies according to the particular needs of each *Casa* but they are generally scheduled in shifts so that there is always at least one TBA on duty. In some of the *Casas* other traditional providers participate. In San Mateo del Mar, both a male and a female traditional healer participate. This is the only site where a male community member participates.

### Linguistic and cultural congruence

The women that collaborate in the *Casas* are all speakers of the local indigenous language, which facilitates communication with the patients. Language congruence is considered to be the primary success by *Casas* personnel. The workshops and training sessions are provided by the coordinators and/or promoters in Spanish, as well as in indigenous languages. Patients perceive that they receive unfair and discriminatory treatment in hospitals, which is one of the reasons they decide to come to the *Casas*, where they feel well cared for by women who speak their language:

*“…Well, I was happy with all of the care I was given…I got along well with the personnel because they spoke Tzotzil and I also speak Tzotzil. We have a good experience with them because you know, when we go to a hospital they don’t treat you the same as here in the Casa, because they speak Spanish and here we don’t speak Spanish. That’s why…” (Female Patient in Chiapas)*.

### Violence prevention and victim services activities

The *Casas* conduct domestic violence prevention activities and organize talks to raise consciousness among authorities and the general public (women and men). The concern with preventing domestic violence is linked to health and the negative effects on the family, as demonstrated in the following quote:

“…It’s not that I’m telling you not to hit, if that’s what you’re accustomed to, well then that’s your custom. What I want is that the women don’t die. That’s my goal in Chalchihuitán. Because look, (…) if the woman dies the children remain, and they’re left alone with the husband. Who will feed them? Who will take care of them? Sometimes another woman comes along, but it’s not the same. That’s the only thing I want, for the women not to die, because the children remain, they grow and they grow up sadly, and [they] won’t be able carry the firewood (for the stove)…” (Coordinator).

Through the work of the *Casas*, domestic violence has gained visibility in the municipal arena and it is now found on the local public agenda in certain municipalities. In addition, the *Casas* have followed up on several concrete cases of violence against women, which has contributed to increasing trust among women who come to the *Casas* in need of services:

“…there are thousands of cases in which they have had an impact, although slight, for example, to speak of violence in a place where the violence is terrible (…). What the women talked about was violence. What they told us was that they wanted a better legal process, because ‘we went to complain and we wound up punished’, so it’s very complex…” (Counselor).

Women’s empowerment has been strengthened by these shifts in referral procedures, both among the women who work in the *Casas* as well as the women they served:

*“The truth is that I’ve talked with them, told them not to allow themselves to be treated that way, because before, before we put up with the hitting. It even happened to me and I was one of them. I didn’t even tell my family that it was happening to me. I was being hit and I didn’t even tell my sisters. Why (not)? …Since I talked with them, I stopped being frightened and my children too. They say ‘it’s okay mom, because you can defend yourself (survive) on your own’ Even the ‘powerful’ (in reference to people that hold a high social position) said to me one time, ‘Señora…you are a woman and you’re not afraid?’ Why do I have to be afraid? I’m not afraid of anyone.” (Health Promoter)*.

### Sexual and reproductive health (SRH) care activities

SRH activities are the most comprehensive services provided in the *Casas* model, and include labor, delivery, and perinatal care. The TBAs are an important resource for culturally responsive reproductive health care. TBAs that work in the *Casas* receive training on referral procedures for women with obstetrical complications; in such cases they accompany the woman to the Western hospital and provide follow up care. Talks on signs and symptoms of obstetric emergencies are also given to the women served. The husband is given an orientation as well so that he can provide support in the post-natal care of the mother and child, and be aware of any symptoms that signal complications.

### Stakeholder perceptions of the *Casas*

The women who work at the *Casas* consider themselves as fulfilling their job responsibility and having a positive effect, exemplified by the indigenous community knowing who they are, what services they offer, and seeking help when needed. The women perceive the greatest achievements to be centered around the fact that patients’ lives have been saved and the level of consciousness among both men and women has increased greatly due to the workshops delivered in community settings that have been well received as evidenced by high attendance, often beyond the expectations of the organizers. Also mentioned is the increase in hospital transfers related to obstetric care, which represents a tangible outcome towards decreasing maternal mortality in the municipalities where *Casas* are located. Furthermore, TBAs are perceived to attend more births in the *Casas* than the governmental health services, playing a vital role in coverage. Another aspect related to the effect of the *Casas* is related to the observed changes in awareness and understanding of problems related to violence, and sexual and reproductive health among the women. Their active participation in findings solutions to these problems is identified as a process of empowerment for the women beginning with the basic recognition of their rights.

However, other stakeholders, such as public servants, were unaware that the *Casas* model even existed, and as a consequence, were unable to consider the value of the services offered. Among the non-indigenous population and those who are not patients, the *Casas* have limited visibility, are perceived as providing very limited coverage, and instead of the growth identified by the women, is perceived to actually suffer from a decrease in productivity specifically related to pre-natal and obstetric care:

“…when I had just arrived, it looked like they were attending more births, seeing more pre-natal patients (…) due to problems or due to the fact that older TBAs have left, well the women don’t come back to seek services, they stay n their communities. It’s almost like I am watching the volume of care decrease” (Medical provider).

## Discussion

Perceptions of the processes and performance of the *Casas* services vary depending on the perspective of informants. Among external collaborators (social services, local authorities, NGOs), achievements and scope of the activities that the *Casas* provide are minimized. This perception can be understood within the context of social inequity, including exclusion, prejudice, discrimination, racism and stereotyping facing indigenous groups, in addition to a perception of the services the Casas provide as being threatening to other service providers. This minimizing of performance and scope by external actors is juxtaposed with the women who collaborate in the *Casas* highlighting their contribution in the detection of emergencies, problems of violence and empowerment of other women from indigenous communities. The women that staff the *Casas* are aware and critical of the gender inequality, exclusion and discrimination to which they have historically been subjected. They view the *Casas* as a genuine opportunity for growth and self-realization as both women and professionals [29].

Moreover, the linguistic and cultural congruency of the *Casas* has been fundamental in light of common care-seeking behavior. Respect for the care-seeking preferences identified by Espinosa’s comprehensive report [9] on the matter were observed during the project evaluation. These preferences included seeking out local resources and the care of TBAs for a variety of reasons: cultural (they speak the same language), effective (they trust they are skilled providers), economic (they charge less), accessibility (they make house calls), and gender (they have experienced childbirth).

The analysis revealed that the processes of the *Casas* transcends sexual and reproductive health care and cases of violence, and speaks to change within larger structural determinants of health equity. Implicitly, the work of the women who have participated in this project, from different places and at different moments, has contributed to strengthening and claiming of indigenous and gender equity, rooted in human rights.

However, the *Casas’* processes and performance are currently limited due to significant organizational and administrative obstacles:

• Lack of formality in the labor relationship that the women have with the *Casas*.

• Lack of explicit structure of and rules for sharing of responsibility among collaborating organizations, which would tailor the organization of the *Casas* according to local needs and resources.

• Lack of clarity about sources of financing, funds, and budget administration. An institutional, bureaucratic approach supported by the public sector turns out to be of little use because the personnel are unable to obtain the receipts necessary to be reimbursed for their expenses. The other approach has been the direct delivery of cash advances to the workers; however, this arouses suspicions and is seen as lacking transparency. Administration of financial resources should be integrated into the process of empowering the women in order to address the aforementioned issues.

• There is currently no formal registration system of cases, which is necessary for providing follow-up care and to contribute in process evaluation.

**Figure 1:**
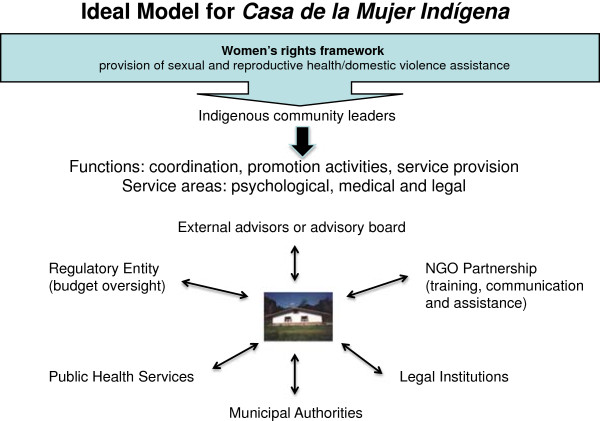
**Ideal model for *casa de la mujer indígena.***

Based on the analysis of the information, an ideal model for the *Casas* was presented to stakeholders in the five sites evaluated (Figure 1).

While this model was designed within a Mexican indigenous context, the structural and functional elements of the model are applicable and relevant to other indigenous contexts. The following elements (as seen in Figure 1) may be useful in settings aiming to provide community-based, culturally responsive health care services to indigenous women, with a focus on provision of services relating to sexual and reproductive health and domestic violence.

a) Community ownership and participation: Based on the experience of the *Casas* we recommend the identification and empowerment of a group of indigenous women who are community leaders to be integrated into the organization. With support from formally trained service providers, they can coordinate activities; train local indigenous participants (for example, TBAs); and offer assistance both in sexual and reproductive health and in cases of domestic violence.

b) Inter-agency partnership and networks: A collaborative relationship with a non-governmental organization should be established to provide support and reinforcement to the work of the indigenous women. There should also be a formal connection with health and legal institutions for follow up on activities related to sexual and reproductive health and domestic violence. We recommend establishing collaborative ties at the local, regional or state levels to strengthen organizational reach.

c) Budget oversight: A sufficient, permanent budget provides continuity to activities and improves performance. Activity and expense reports should be submitted to the funding agency for transparency and accountability.

d) External advisor or advisory board: Responsible for supporting training activities and generating applicable evaluation and research agendas.

## Conclusions

Although few similarities were observed between the project’s original model of service delivery and regionally specific implementation, the qualitative evaluation of this model of care noted important positive processes and performance at different levels. Most notably, the Casas facilitated indigenous women’s access to referrals to other institutions for emergency obstetric care and in cases of violence. The work of the community promoters increased the number of births in health centers and regional hospitals as well as legal referrals and the filing of domestic violence complaints with local authorities. An increasing number of obstetric emergency cases are now referred to regional hospitals or health services, which demonstrates the importance of the *Casas* in the detection of obstetric emergencies as a formal point of referral. These accomplishments are significant and demonstrate the possibilities of changing longstanding inequity in access to care via community-based service delivery.

The current sustainability of this project is based on the dedication and voluntary work of the women that collaborate in the *Casas*, and their willingness to work without a regular schedule or salary. The *Casas* are considered an organized response in confronting the context of social and economic inequality in which indigenous women are immersed. The *Casas* model successfully facilitates access to effective and culturally appropriate health care within a context of organizational vulnerability and social inequity. Strengthening of the model, and regular monitoring and evaluation of the work are important contributions to achieving equitable access to quality care for rural indigenous populations.

## Endnotes

^a^By the term “indigenous” reference is made to populations that descend from native peoples of a territory that was later settled.

^b^More than 10% of the national population, approximately 12.7 million Mexicans, belong to at least one of the more than 62 clearly identified ethnic groups. Mexico has the largest indigenous population of the Americas.

## Competing interests

The authors declare that they have no competing interests, financial or otherwise.

## Authors’ information

BPV is a social psychologist and researcher with the Center for Health Systems Research at Mexico’s National Institute of Public Health. She studies health conditions in indigenous populations, specifically among women and older adults. MR is a public health researcher with the School of Medicine, Department of Public Health at the Industrial University of Colombia. She studies health outcomes and service utilization among vulnerable populations, particularly displaced/refugee communities. SM is an medical doctor with training in anthropology and public health. He is with the Regional Center for Public Health Research at Mexico’s National Institute of Public Health. He studies health systems and public policy in indigenous populations in Mexico. CA is a nutrition researcher with the Center for Research in Nutrition and Health at Mexico’s National Institute of Public Health. She studies health behavior and nutritional outcomes. MM is a public health researcher with the Center for Health Systems Research at Mexico’s National Institute of Public Health. She studies the health of older adults, as well as health promotion. KC is a visiting professor in the Department of Biology at Fort Lewis College where she teaches social science and health science courses. AT is an independent consultant for public health research and evaluation projects. Her expertise is in reproductive health, qualitative methods, and health services for vulnerable populations in Mexico and the U.S.

## Authors’ contributions

BPV contributed with the original idea and design of the study, coordinating the field work, analysis and interpretation of the data, and writing the drafts and final version of the manuscript. MR contributed to the discussion of study design, field work in Puebla, analysis and interpretation of the data, drafting the manuscript and approval of the final version. SM contributed to the discussion of study design, field work in Oaxaca and Chiapas, analysis and interpretation of the data, drafting the manuscript and approval the final version. CM participated in field work in Oaxaca and Guerrero, analysis and interpretation of the data. MM participated in the discussion of study design field work in Oaxaca and Guerrero, analysis and interpretation of the data, drafting the manuscript and approval the final version. KC and AT participated in writing the drafts and final version of the manuscript, and preparation of the manuscript for publication. All authors have approved the final version of the manuscript.
